# Design theory of compact power divider with reconfigurable power division and negative group delay characteristics

**DOI:** 10.1038/s41598-023-34272-y

**Published:** 2023-05-04

**Authors:** Rekha G. Nair, Natarajamani S

**Affiliations:** Department of Electronics and Communication Engineering, Amrita School of Engineering, Coimbatore, Amrita Vishwa Vidyapeetham, Coimbatore, India

**Keywords:** Engineering, Mathematics and computing

## Abstract

This article presents the combined analysis of reconfigurable power division and negative group delay (NGD) in a power divider. A novel composite transmission line based reconfigurable power divider with high power division ratio, variable negative group delay, and lower characteristic impedance is presented in this work. The impedance transformation in composite transmission lines control both negative group delay and power division. This power divider possesses a wide range of power division ratios from 1 to 39, adequate isolation, impedance matching, and NGD of $-3.4$ ns to $-4.7$ ns in the reconfigurable transmission path. The negative group delay is achieved without using any additional group delay circuits. Theoretical equations corresponding to the low characteristic impedance of the transmission line sections and that of isolation elements are derived. The measurement results justify the attainment of high tuning of the power division ratio and negative group delay. Isolation and return loss are higher than − 15 dB at the centre frequency of 1.5 GHz. The significant contributions of this design can be listed as the wide reconfigurable power division along with negative group delay and reduced size.

## Introduction

Wireless communication systems require equal, unequal, and reconfigurable power dividers as feeding networks for the antenna arrays. In reconfigurable power dividers operating band or power-dividing ratio is controlled by DC voltages and lumped elements like varactor diodes. The capacitance of varactor diodes can be controlled by DC voltage, resulting in bandwidth or power-dividing ratio change^1–3^. In addition to reconfigurability, negative group delay (NGD) is also required to overcome beam squint problems in antenna array elements. Some reconfigurable power dividers are presented in^4–7^. Switchable inverters are used for getting fully reconfigurable power divisions in^4^. However, the presence of twenty PIN diodes makes the system complex. In^5^, only two varactor diodes are used, but tunability is from 1:1 to 1:2.4 only. Reconfigurable PDs with continuous division ratio are designed in^6^, but the power division range of $-12.4$ to 14.8 dB and $-8.6$ to $-22.5$ dB only. PD with a more comprehensive division from $-1.25$ dB to 20 dB is realized in^7^, but the circuit uses a large number of different characteristic impedances. Planar magic-T-based voltage-controlled variable power divider with a maximum power division range of 14 dB is presented in^8^. However, the circuit has a phase difference of around 15 degrees. Power splitting architecture of SWIPT system is studied in^9,10^ and reconfigurability based on cascaded varactor diode and a reflective phase-shifter method is presented in^11–13^. The dual band operation  and isolation section with additional transmission lines is shown respectively in^14,15^ to eliminate the undesirable coupling occurring while tuning.

Phased array antennas suffer from beam-squint problems, which leads to unwanted perturbation in the shape and direction of the radiation pattern. A power divider with tunable PDR and NGD will be beneficial to overcome this design challenge^16^. Power dividers based on controlling insertion loss and having a wide range dividing ratio and narrow bandwidth are presented in^17^. However, GD analysis is not done in this work. The design of a negative group delay filter with 4.05 ns negative group delay is implemented in^18^. A systematic analysis of the NGD power divider is done in^19^. The concept of cascaded identical second-order baseband stages is utilized in this work. A balanced-to-unbalanced negative group delay power divider based on short-circuited coupled lines with resistors is presented in^20^.

The conventional power dividers designed for antenna arrays using negative group delay circuits suffer from small fractional bandwidths (FBWs) and poor reconfigurability in power division ratio (PDR). Combining power division reconfigurability and negative group delay will compensate for circuit group delay. In RF circuits, the negative group delay phenomenon is observed within a narrow frequency band, it will increase linearity resulting in enhanced performance of a wireless communication system^21^ as in the case of squint-free series-fed antenna arrays. A distributed passive bandpass negative group delay circuit cascaded with a microwave amplifier is presented in^22^. The mathematical equations for calculating group delays associated with the magnitudes of transmission coefficients at a power division ratio $k^2$ for designed operating frequency is given in^23^.

All works mentioned earlier, are successful in realizing functions like tunable bandwidth, frequency, power division, or NGD. A work simultaneously addressing power division reconfiguration and negative group delay is not yet reported in literature.

This article presents a new design technique to achieve negative group delay and tunable power division ratio without additional group delay circuits. The impedance transformation in composite transmission lines controls the negative group delay and power division. In the proposed circuit, the transmission path between 3 and 1 provides negative group delay characteristics, and positive group delay is provided in the transmission path between 2 and 1. The proposed power divider can be used in envelope-tracking applications or dynamic power amplifiers. The positive group delay path can be directly linked to the RF path, and the negative group delay path can be connected to the detector path to make up for the time mismatch between the signal envelope and the dynamic power supply. The circuit also maintains proper impedance matching and adequate isolation. The paper is arranged with the theoretical analysis and design techniques in “Design techniques and mathematical analysis” Section, and “Analysis of results” Section analyzes and investigates results in different scenarios and compares this work with the recent state-of-the-art designs in the literature. The work is then concluded in “Conclusion” Section.

## Design techniques and mathematical analysis

**Figure 1:**
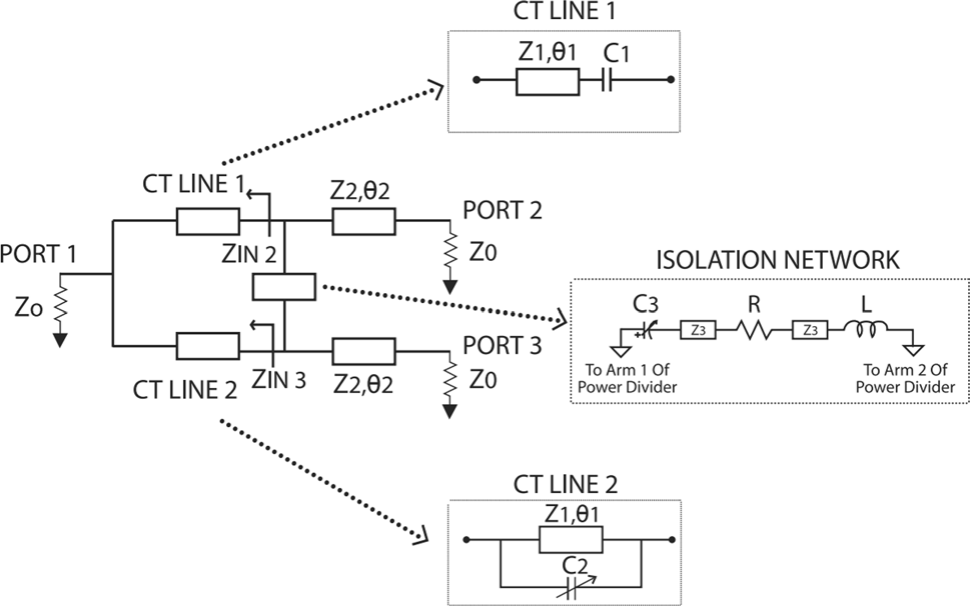
The block diagram of power divider with reconfigurable PDR.

The concept of composite transmission lines (CT lines) is used in this design to attain: reconfigurability in power division, smaller characteristic impedance, size reduction  and negative group delay. The isolation network has additional transmission lines and lumped elements to improve port matching and reconfigurability. The block diagram of the proposed power divider is shown in Fig.1. The transmission matrix of a conventional quarter wavelength (QWL) section is equated to that of the power divider’s modified branches (CT lines). The power flowing through the dividing arms depends on the impedance of the arms. The arm with smaller impedance will take more current, and therefore more power.

The relationship between composite transmission lines and quarter wavelength line can be expressed in matrix form as:

$$\begin{aligned}{} & {} \begin{pmatrix} M_1 \\ \end{pmatrix} \ * \begin{pmatrix} M_{C_1}\\ \end{pmatrix} = \begin{pmatrix} M_{QWL}\\ \end{pmatrix} \\{} & {} \begin{pmatrix} Cos\theta _1 &{} jZ_1 Sin\theta _1 \\ jY_1 Sin\theta _1 &{} Cos\theta _1 \end{pmatrix}*\begin{pmatrix} 1 &{} - \frac{j}{\omega C_1}\\ 0&{} 1 \end{pmatrix}= \begin{pmatrix} 0 &{} - {j}{\sqrt{2}Z_0}\\ \frac{j}{\sqrt{2}Z_0} &{} 0 \end{pmatrix} \end{aligned}$$

$M_1$ is the transmission matrix of a microstrip transmission line and $M_{C1}$ and $M_{QWL}$ are that of capacitor and quarter wavelength line respectively. By solving the matrices the value of tuning element ( $C_1$) can be found.

**Figure 2:**
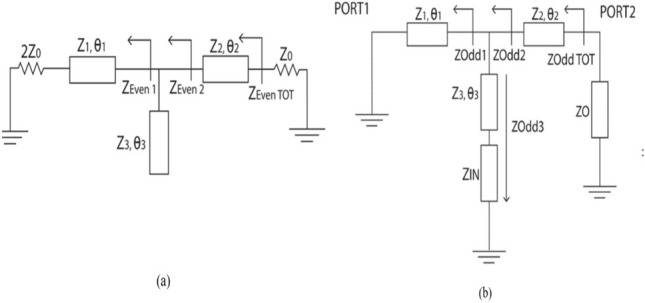
Equivalent Circuits (**a**) Even mode (**b**) Odd mode.

The equivalent circuit model of the PD for even mode analysis is shown in Fig.2a and based on that we get (1), (2), (3) Here $\theta _1$, $\theta _2$ and $\theta _3$ represent the electrical length and $Z_1$, $Z_2$, and $Z_3$ corresponds to characteristic impedance.

1 $$\begin{aligned} Z_{Even1}= & {} Z_{1} \frac{2Z_{0}+j2Z_{1}tan\theta _{1}}{Z_{1}+j2Z_{0}tan{\theta _{1}}} \end{aligned}$$

2 $$\begin{aligned} Z_{Even2}= & {} \frac{{Z_{Even1}}{Z_{Even3}}}{Z_{Even1}+Z_{Even3}} \end{aligned}$$

where, $$Z_{Even3}$$ = $$Z_{3/jtan} \theta _3$$

3 $$\begin{aligned} Z_{EvenTOT}= Z_{2} \frac{Z_{Even2}+jZ_{2}tan\theta _{2}}{Z_{2}+jZ_{Even2}tan{\theta _{2}}} \end{aligned}$$

For impedance matching LHS of (3), can be equated to port impedance $Z_0$ , giving:

3a $$Z_{0} = Z_{2} \left[ {\left( {Z_{{Even2}} + jZ_{2} \tan \theta _{2} } \right)/\left[ {Z_{2} + jZ_{{Even2}} \tan \theta _{2} } \right]} \right]$$

Modifying (2) using (1) and later applying in (3a) leads to the values of $Z_2$ and $Z_3$.

4 $$\begin{aligned} Z_2= & {} \sqrt{\frac{{(2Z_{0}^2+Z_1^2+4(Z_0^2/P))-(P2Z_0^2) -(PZ_1^2)}}{2+2/P}} \end{aligned}$$

5 $$\begin{aligned} Z_3= & {} \frac{Z_1 Z_{2} tan\theta _3[2Z_2tan\theta _{2} - Z_1tan\theta _1]}{Z_1 Z_2 + (Z_1^2 - 2Z_2^2)tan\theta _{1} tan\theta _2} \end{aligned}$$

Here P = $tan^{2}\theta _1$. Based on the circuit given in Fig. 2.b odd-mode analysis is done to find the values of lumped elements in the isolation circuit. $Z_{IN}$ in the circuit corresponds to the impedance of the isolation network. So, we get:

$$Z_{Odd1}$$ = j $$Z_{1} tan\theta _1$$

and

6 $$\begin{aligned} Z_{Odd3}= & {} Z_{3}\left[ \frac{(RLC_{series}/2) +jZ_{3}tan\theta _3}{Z_{3}+j(RLC_{series}/2)tan\theta _3} \right] \end{aligned}$$

7 $$\begin{aligned} Z_{Odd2}= & {} \frac{Z_{Odd1}.Z_{Odd3}}{ Z_{Odd1}+Z_{Odd3}} \end{aligned}$$

Substitute for $Z_{Odd3}$ and $Z_{Odd1}$ in (7). The modified value of $Z_{Odd2}$ is then applied in (8), which leads to (8a). On separating the real and imaginary parts of (9) after this modification gives (10).

8 $$\begin{aligned} Z_{ODDT\,OT} = Z_{2}\frac{ Z_{Odd2}+jZ_{2tan\theta _2}}{Z_2+jZ_{Odd2}tan\theta _2} \end{aligned}$$

8a $$\left( {Z_{{ODDT\,OT}} = \left[ {Z_{{Odd3}} /2\sin ^{2} \theta _{1} } \right] + \left[ {{\text{j}}Z_{2} /\tan \theta _{2} } \right]} \right)$$

$Z_{Odd3}$ in isolation network consists of added transmission line section $Z_3$ and series RLC. So $Z_{ODD3}$ term in (8a) is modified using $Z_3$ and series RLC resulting in (9).

9 $$\begin{aligned} Z_{ODDT\,OT}= \frac{2Z_{3}+R}{2sin^{2}\theta _1} +j \left[ \frac{\omega L - \frac{1}{\omega C_3}}{2sin^{2}\theta _1} +\frac{Z_2}{tan\theta _2} \right] \end{aligned}$$

To get perfect matching the real part can be equated to $Z_0$ and imaginary part to zero, this results in (10), (11)

10 $$\begin{aligned} R=Z_0*2Sin^2\theta _1-2Z_3 \end{aligned}$$

and

11 $$\begin{aligned}{}[\omega L] - [1/\omega C_3] = 2Z_2 \frac{sin^{2}\theta _1}{tan\theta _2} \end{aligned}$$

The criteria for matching is that the sum of the impedance of the composite transmission lines should be equal to the impedance of the bridge separating the arms, $(Z_{Odd3}=Z_{IN2}+Z_{IN3})$. Substituting for $Z_{IN2}$ and $Z_{IN3}$ based on CT lines shows that $C_2=2C_3$. The impedance of the lower CT line can be transformed w.r.t. reconfigurable isolation network based on this condition, and this results in different ratios in the power division.

The group delays associated with the magnitudes of transmission coefficients at a power division ratio $k^2$ and operating frequency f can be evaluated based on Eqs. (12) and (13),

12 $$\begin{aligned} \tau _{21}= & {} \frac{1+2k^2 +\sqrt{1+k^2}+k^2\sqrt{1+k^2}}{8fk^2\sqrt{1+k^2}} \end{aligned}$$

13 $$\begin{aligned} \tau _{31}= & {} \frac{1+2k^2}{4f2k\sqrt{1+k^2}} \end{aligned}$$

### Design procedure

The proposed design is based on the concept of composite transmission lines. The quarter wavelength lines in a conventional Wilkinson based power divider are replaced by composite transmission lines. Reconfigurability is attained by varying the impedance of the composite transmission lines. The initial value of the capacitance in the composite transmission lines is found by an analysis based on the transmission matrix relationship between the quarter wave length line and composite transmission line . Even mode analysis is used to find the characteristic impedance of the transmission line sections. These impedance values are evaluated as per even mode analysis. Odd-mode analysis is used to find the values of lumped elements in the isolation circuit.

**Figure 3:**
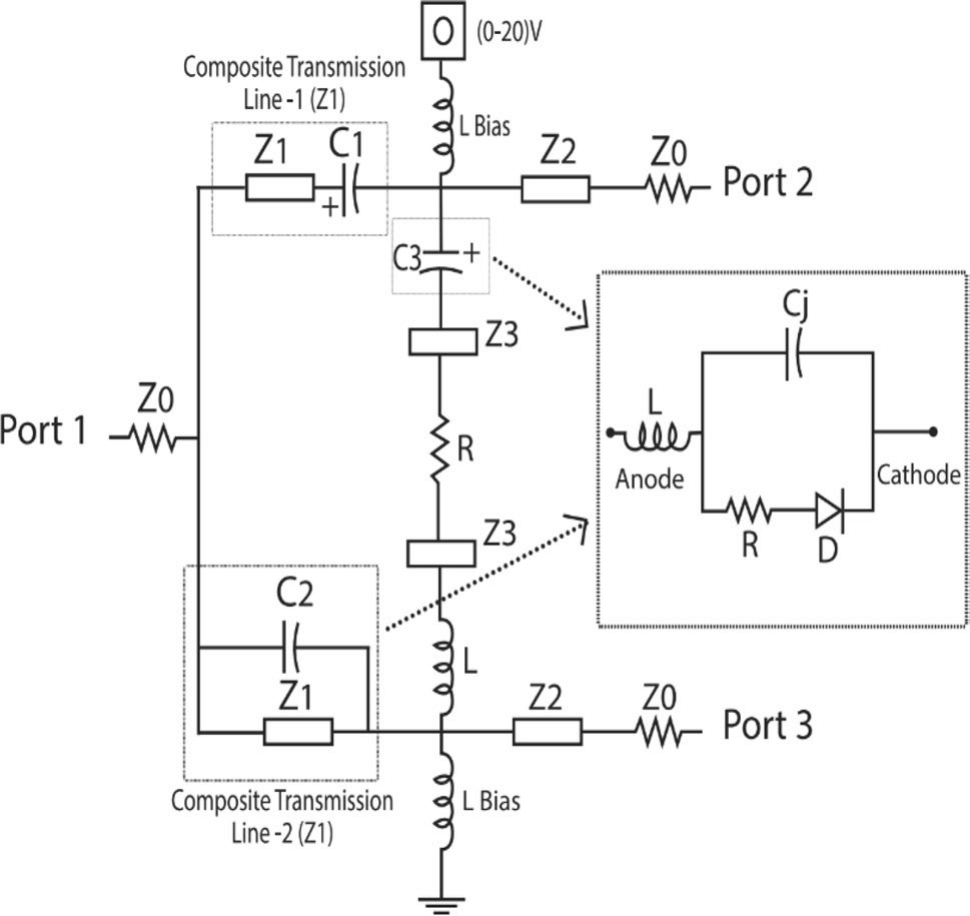
Schematic of the design.

The schematic of the design is shown in Fig.3. It has three different transmission line sections: $Z_1$, $Z_2$, $Z_3$, and an isolation network. Impedance $Z_1$ corresponds to the characteristic impedance of composite transmission lines placed in the dividing arms. Tuning the varactor diodes associated with composite transmission lines results in transforming the corresponding impedance and hence the power flowing through the dividing arms. Based on even mode analysis the characteristic impedance $Z_2$ and $Z_3$ are found. Here $Z_2$ is the impedance of transmission line sections leading to the output arms, and $Z_3$ is that of the extended transmission line sections placed in the isolation arm. These values are given by Eqs. (4) and (5). Odd mode analysis is done to find the values of the lumped elements in the isolation arm. The lumped element values are found using Eqs. (10) and (11). The design parameters are listed in Table 1. The group delay associated with output ports can be calculated from (12) and (13).

**Table 1:** Design parameters and dimension.

Impedance (ohms)	Electrical length (degrees)	lUMPED elements	Length (mm)	Width (mm)
$Z_1$ = 65	55	R = 150ohms	$\hbox {L}_0$ = 2.5	$\hbox {W}_0$ = 2.2
$Z_2$ = 60	30	L = 3.3nH	$\hbox {L}_1$ = 15.5	$\hbox {W}_1$ =1.51
$Z_3$ = 45	7	$\hbox {C}_1$ = 4.7pF	$\hbox {L}_2$ = 8.56	$\hbox {W}_2$ = 1.51
		C2,C3 (VD = SMV2019)	$\hbox {L}_3$ = 1.87	$\hbox {W}_3$ = 1.51

The design procedure is summarized as follows:

1. Specify the centre frequency f and PDR.
2. Replace the conventional quarter wave length lines by composite transmission lines, based on the value of $Z_1$ and lumped elements obtained by solving the transmission matrix.
3. Find the values of impedance $Z_2, Z_3$ as per (4) and (5).
4. Determine the value of components present in the reconfigurable isolation network as per (10) and (11).
5. Realize the capacitors $C_2$ and $C_3$ by varactor diode SMV 2019LF from Skyworks solutions.
6. Calculate GD at output ports as per (12) and (13).
7. Vary the biasing of varactor diodes (VD) to vary the capacitance in the ratio $C_2=2C_3$ and hence vary the arm impedances which in turn results in variation in PDR and NGD.
8. Compare the attained and calculated results.

## Analysis of results

The prototype is fabricated with a Roger RT/5880 substrate having $\epsilon _r$ = 2.2 and a height of 0.8 mm. The proposed work is based on the impedance transformation of the composite transmission line sections. The composite transmission line sections are having low impedance than quarter wavelength transmission lines and hence overcome the fabrication limitations. By transforming the impedance of the composite transmission line, we can vary the power flow through the line. This controls the insertion loss (IL) in the pass band. The ratio of the power available at the output ports can be mathematically calculated based on the magnitude of insertion loss. While tuning, the arm of the power divider corresponding to port three significantly affects its impedance. So, controlling the impedance using varactor diodes results in variation in the power-dividing ratio. When equal power is needed at output ports, the varactor diodes are placed in destructive mode, i.e., unbiased. The R and L values of the isolation network are calculated as R = 120 ohms and L = 3.3 nH to attain perfect isolation at the center frequency. A power divider with a power division ratio of 1:39, 30 percentage size reduction, and a transmission path with reconfigurable negative group delay is designed at a center frequency of 1.5 GHz.

**Figure 4:**
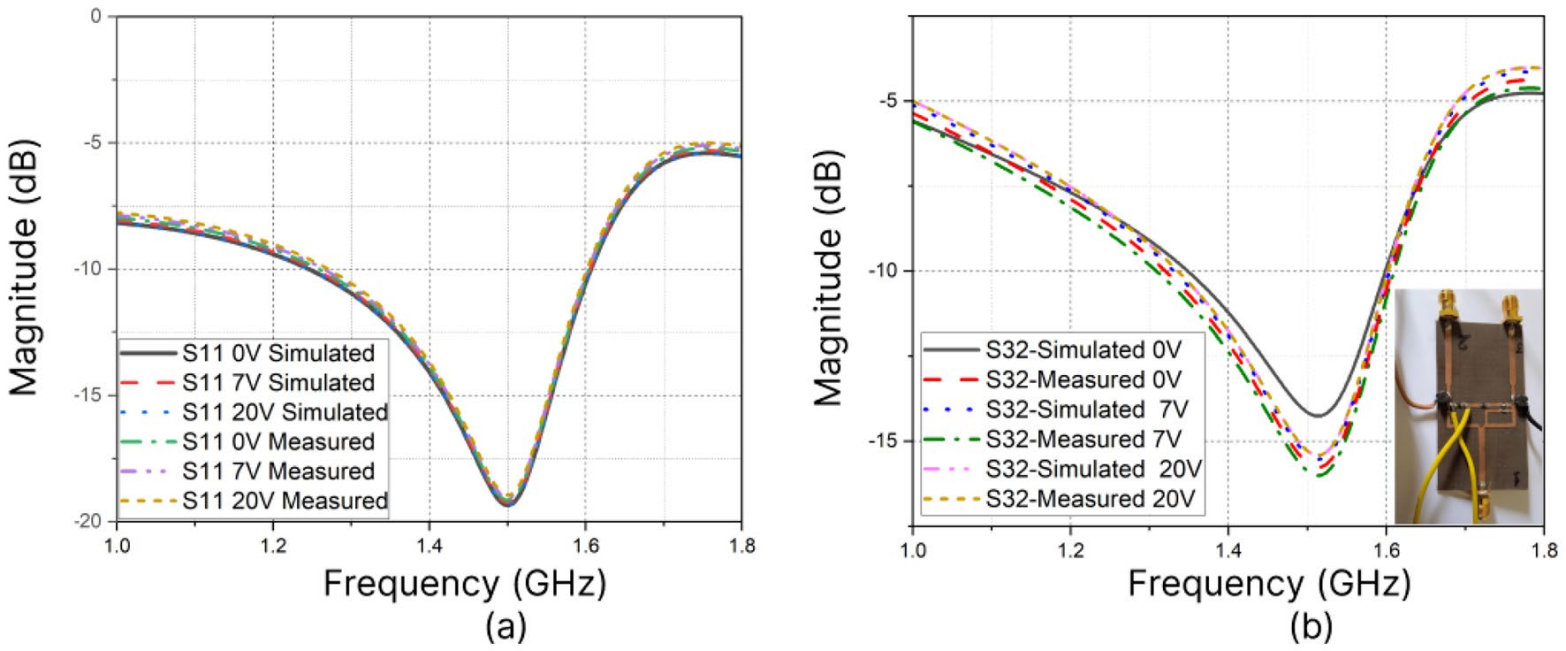
(**a**) S11 at different biasing voltages (**b**) S32 at different biasing voltages.

**Figure 5:**
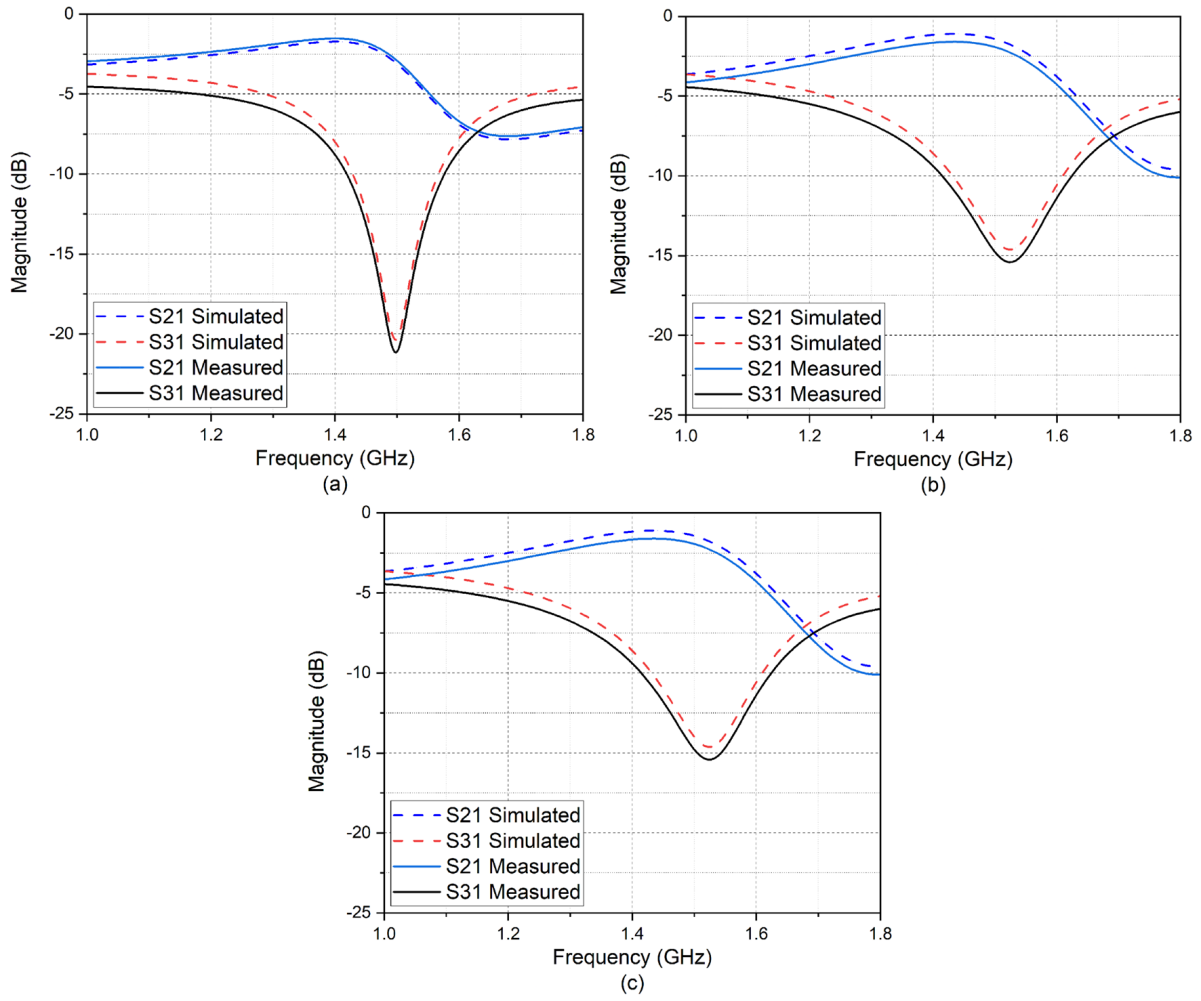
Variation in PDR w.r.t. biasing voltages: (**a**) PDR = 39 (Vdc = 0 V) (**b**) PDR = 15 (Vdc = 7) (**c**) PDR=7 (Vdc = 20 V).

The simulation is carried out using ANSYS-HFSS software. Hyper abrupt junction varactor diode SMV 2019LF is used for tuning the capacitance $C_2$ and $C_3$. Tuning the varactor diodes for different biasing voltages provides impedance transformation, which in turn results in reconfigurability in power division and NGD. Transmission line sections with short electrical length in the isolation network compensate for the limited capacitance range of commercial varactor diodes. This improves the overall tuning range. The biasing voltage across the varactor diode is applied through RF choke inductors. The schematic of the designed PD is shown in Fig 3. The performance of the circuit is validated by measuring the scattering parameters and group delay using a Keysight E5C vector network analyser. When the control voltage changes from (0–20 V), the capacitance of the varactor diode varies from (2.2–0.3) pF. This transforms arm impedance and results in a variation in power flow from − 1.47 dB to − 16.4 dB, resulting in a Power division ratio up to 39. The simulated and measured input return loss is around − 19 dB at the centre frequency and is almost constant regardless of the tuning of the capacitance values. Tuning of the varactor diodes without altering the impedance matching accounts for this. Isolation also is greater than − 15 dB at the centre frequency (1.5 GHz). Figure4a shows the magnitude of S11, and Fig. 4b shows the magnitude of isolation (S32) for different biasing voltages corresponding to different power division ratios. The power division ratio at the output is calculated mathematically from the results of insertion loss. The measurement and simulation results for power division corresponding to three different power division ratios are illustrated in Fig.5. Power division is controlled by using impedance transformation of the composite transmission line; there is a wider range to divide power unequally over a narrow range of DC voltage. So, a (0–20 V) DC voltage source is sufficient.

The fractional power division ratio (FPDR) is a significant performance parameter for power dividers with a reconfigurable power division ratio. The fractional power division ratio can be evaluated from (14) based on the values of the Maximum power division ratio (MPDR) and maximum and minimum values of insertion loss. From fractional power division ratio, the reconfigurability of the proposed power divider can be found as 87 percent. This parameter is not analysed in the case of most of the reported reconfigurable power dividers. The fractional power division ratio value for three different power division ratios is shown in Table 2. The FPDR value can be seen as increasing with the power division ratio.

14 $$\begin{aligned} FPDR= \frac{(MPDR-1) (IL_{max}-IL_{min})*100}{MPDR * IL_{max}} \end{aligned}$$

The analytical study shows that the group delay associated with the transmission paths between 3 and 1 is negative, and that associated between transmission paths 2 and 1 is positive.

**Table 2:** Fractional power divisiion ratio in different cases.

Cases	MPDR	IL_Max_	IL_Min_	FPDR
1.	7	11.1	1.8	78
2.	15	12.2	1.8	80
3.	39	19.6	1.8	87

**Figure 6:**
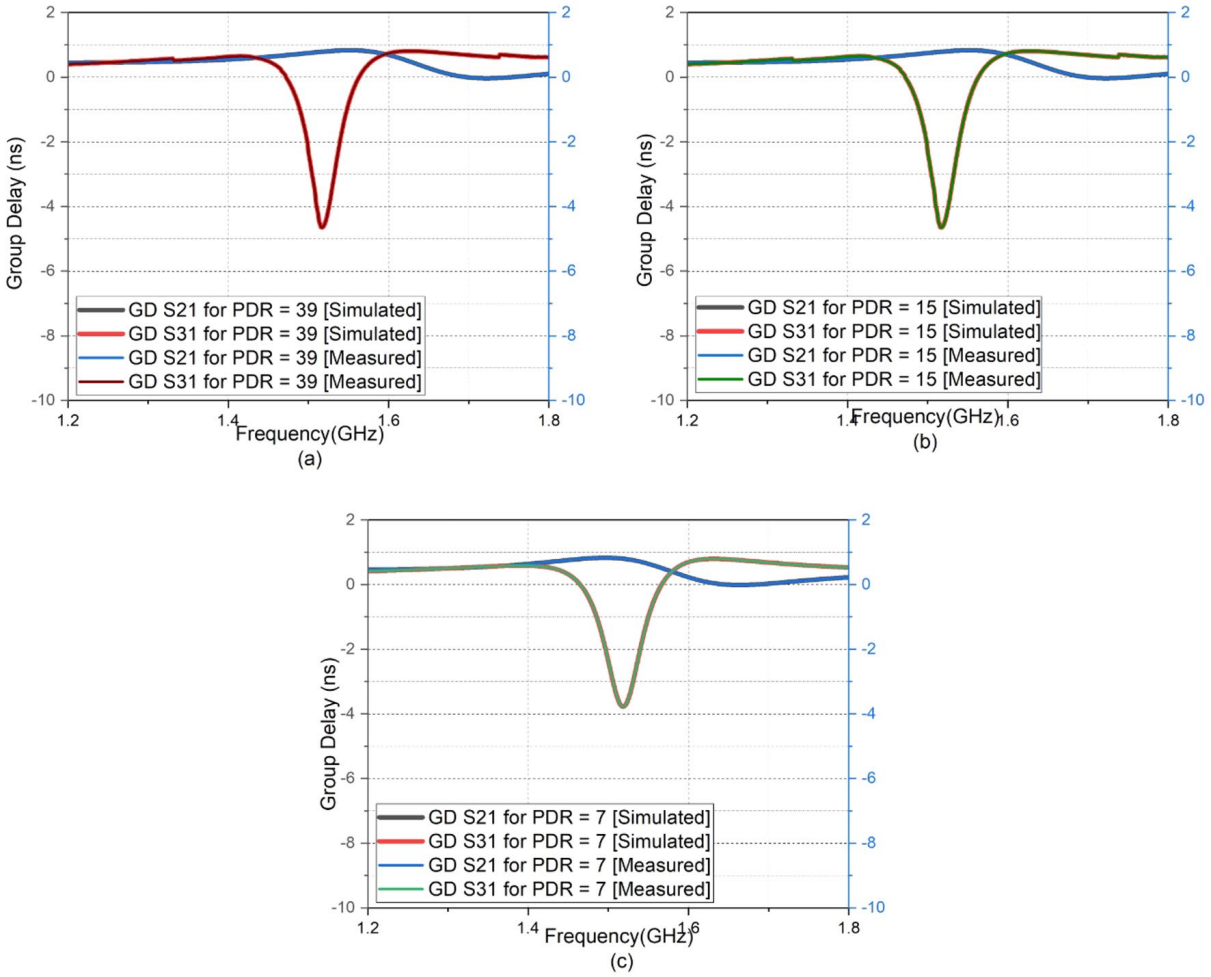
Variation in group delay w.r.t. biasing voltages: (**a**) PDR = 39 (Vdc = 0 V) (**b**) PDR = 15 (Vdc = 7) (**c**) PDR = 7 (Vdc = 20 V).

The distortion level can be kept below the desired level by reducing the effective bandwidth below the 3 dB cut-off. Also, if the negative group delay bandwidth spans over the entire range of frequency response, then the distortion matric will be large in that region. In this work, the negative group delay bandwidth (i.e., bandwidth with$\tau <0$ ) is 100 MHz. The NGD-BW (Negative group delay-bandwidth) product for the three different cases of power division is 0.46, 0.45, and 0.36, respectively for power division ratios of 39, 15, and 7. The negative group delay corresponding to different power division ratios of 7, 15, and 39 are shown in Fig.6a, b, c. This 100 MHz band width can be reduced (to 60 MHz) by transforming the impedance from port 1 to port 3. The NGD*BW product numbers of 0.46, 0.45 and 0.36 hence reduce to 0.28, 0.27 and 0.22.

The transmission path with a series-connected composite transmission line gives positive group delay, and the transmission path with a parallel-connected composite transmission line provides negative group delay. The path of negative group delay can be used to compensate for the non-linearity in time between the signal envelope and dynamic power supply, whereas the path with positive group delay can be linked directly to the RF path. So, the proposed power divider is a promising design that can be used in dynamic power or envelope tracking power amplifiers. The almost constant phase difference observed in a bandwidth of 250 MHz around the centre frequency is an added justification for this application. The graph shows that negative group delay w.r.t different PDRs varies from -3.6 ns to -4.6 ns in paths 1 to 3. Hence path three can be used to make up the non-linearity in time between the dynamic power supply and signal envelope. As the value of the impedance associated with arm three is varied to lower values, the value of S31 and negative group delay also decreases. But group delay at port two and S21 remains almost constant. So, port two can be directly linked to the RF path. The experimental group delay values are verified with the corresponding theoretical values as per Eqs. (12) and (13). The limitation in working bandwidth is due to the trade-off between the operating bandwidth and the maximum achievable negative group delay.

**Figure 7:**
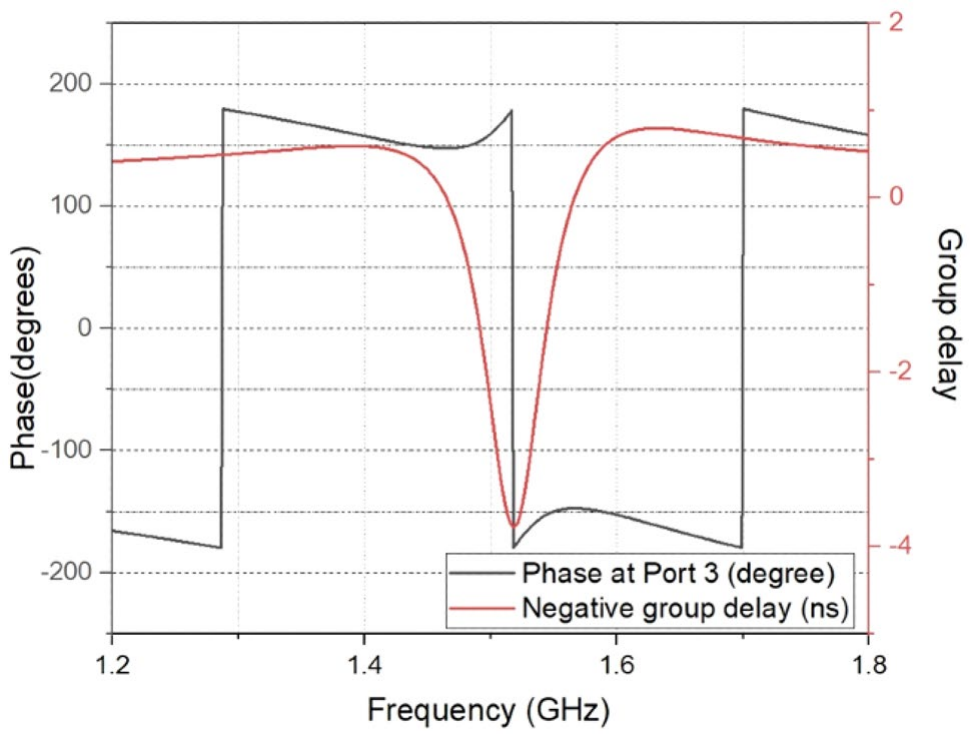
Phase response and negative group delay at port 3.

**Figure 8:**
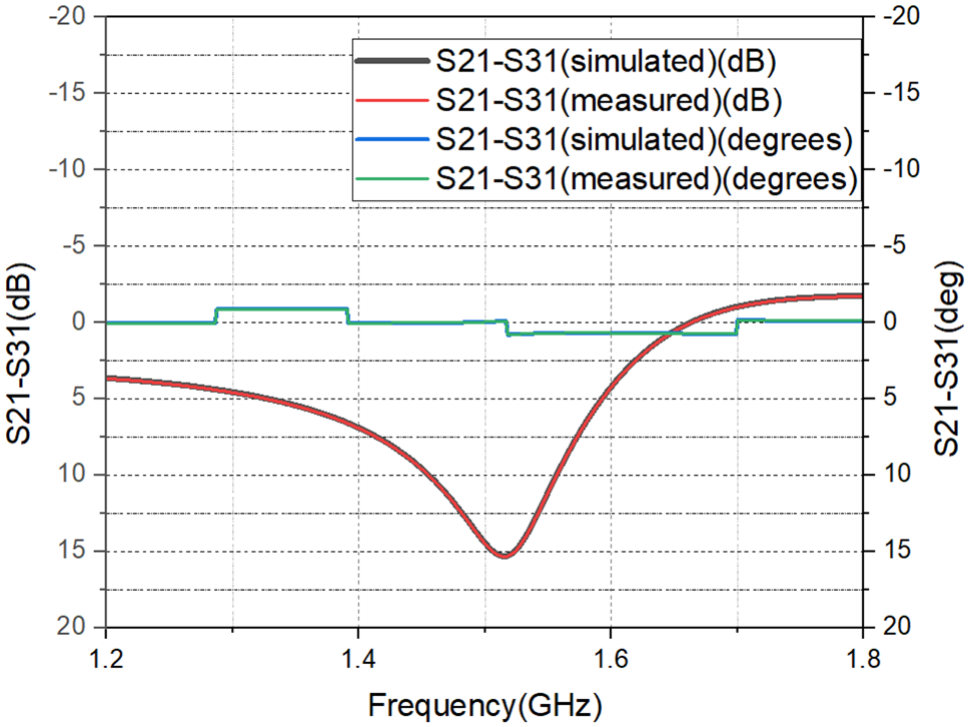
Amplitude and phase imbalance between out put ports.

**Figure 9:**
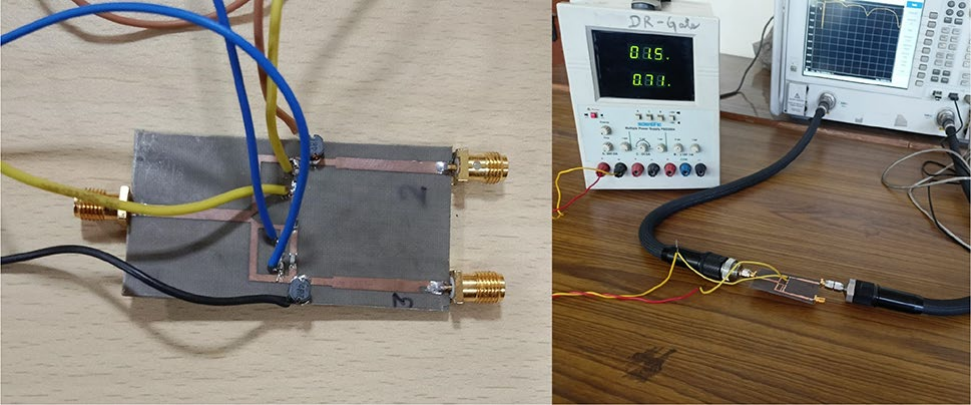
Prototype and measurement set up of the power divider.

The negative group delay and phase at the centre frequency are shown in Fig. 7. The amplitude and phase difference corresponding to a power division ratio of 39 is shown in Fig. 8. The phase difference is around 1.25 degrees only. The unequal power division nature of the circuit accounts for the high amplitude imbalance. The prototype and measurement setup of designed power divider is shown in Fig. 9.

The prototype and measurement setup of the designed is shown in Fig.9.

The novelty of the design technique and the novelty of the application-oriented results are the key features of this design. Table 3 demonstrates the advantages of this design based on a comparison with other recent publications. The table reveals the wide and high PDRs achieved by this design, along with the compact size and negative group delay path without using additional negative group delay circuits.

**Table 3:** Table of performance comparison of the proposed PD with related state-of-the-art power dividers.

Refs.	$f_c$ (GHz)	Size($\lambda _g^2$)	No. of LE, AE	RL (dB), Isln (dB)	IL (dB)	PDR	NGD * BW
^1^	2.4	0.67*0.8	5 and 1	$\ge -20$ , $\ge 12$	− 2/− 14	1:28	NIL
^2^	2.4	0.7*0.81	3 and 2	$\ge -22,-15$	− 2.7/− 4.2	1:10	NIL
^6^	2.45	0.66*0.81	5 and 2	$\ge -20,-20$	− 1.23/− 1.11	1:14	NIL
^13^	2.45	0.6*0.75	5 and 2	$\ge -19$ ,-20	− 1.3/− 0.97	1:29	NIL
^15^	1.2	0.2*0.19	2 and 5	$\ge -20,-15$	− 2.2/− 4	1:1.5	NIL
^19^	2.14	0.47*0.45	NIL (not reconfigurable)	$\ge -35$ , -42	9.29–9.3	1	− 0.174
^23^	2.14	0.57*0.33	NIL (not reconfigurable)	$\ge -11.4$ , -30.4	4.98–7.48	1	− 0.04
Proposed work	1.5	0.38*0.74	3 and 2	$\ge -15,-14$	− 2.1/− 19.4	1:39	− 0.36 to − 0.46

${{*}}{f_c}$ = centre frequency, *LE* = lumped elements, *AE* = active elements, *RL* = return Loss, *Isln* = isolation, *IL* = insertion Loss, *PDR* = power division ratio, *BW* = $\tau < 0$ Bandwidth , *NGD* =negative group delay.

A power divider simultaneously performing reconfiguration of the power division ratio and negative group delay is not yet reported in the literature. Such a power divider is a promising candidate for dynamic power or envelope-tracking power amplifiers. This design can also be used in squint-free antenna arrays. The bandwidth of the proposed design is limited due to the trade-off between the maximum attainable value of negative group delay and bandwidth. Cascading more sections with a slight difference in center frequencies may enhance the bandwidth.

## Conclusion

This paper presents a novel method of designing a reconfigurable power divider with high power division ratios and negative group delay. The high range of power division, the existence of a negative group delay path, lower number of tuning elements, low characteristic impedance (< quarter wavelength lines ), compact size, and high fractional power division ratio are the significant advantages of the proposed design. This design is a novel solution to simultaneously achieve high reconfigurable PDR and negative group delay, which is suitable for dynamic power or envelope-tracking power amplifiers and in squint-free antenna arrays. The design equations, along with experimental results, have been presented. The composite transmission lines reduce the impedance values and help to attain negative group delay without additional group delay circuits. Small electrical length transmission lines in the isolation arm improve the tuning range of varactor diodes.

## Data Availability

All authors are agreeing to share the supplementary files on reasonable request received by corresponding author. The data sets used and/or analysed during the current study are also available from the corresponding author on reasonable request.
